# Association between Melanocytic Nevi and Risk of Breast Diseases: The French E3N Prospective Cohort

**DOI:** 10.1371/journal.pmed.1001660

**Published:** 2014-06-10

**Authors:** Marina Kvaskoff, Anne Bijon, Sylvie Mesrine, Alice Vilier, Laura Baglietto, Agnès Fournier, Françoise Clavel-Chapelon, Laure Dossus, Marie-Christine Boutron-Ruault

**Affiliations:** 1 “Nutrition, Hormones and Women's Health” Team, Inserm U1018, Centre for Research in Epidemiology and Population Health (CESP), F-94805, Villejuif, France; 2 Université Paris Sud 11, UMRS 1018, F-94807, Villejuif, France; 3 Gustave Roussy, F-94805, Villejuif, France; 4 Channing Division of Network Medicine, Department of Medicine, Brigham and Women's Hospital and Harvard Medical School, Boston, Massachusetts, United States of America; 5 Cancer Control Group, QIMR Berghofer Medical Research Institute, Herston, Queensland, Australia; 6 Cancer Epidemiology Centre, Cancer Council of Victoria, Melbourne, Victoria, Australia; 7 Centre for Molecular, Environmental, Genetic and Analytic Epidemiology, School of Population Health, University of Melbourne, Victoria, Australia; Medical School, King's College London, United Kingdom

## Abstract

**Background:**

While melanocytic nevi have been associated with genetic factors and childhood sun exposure, several observations also suggest a potential hormonal influence on nevi. To test the hypothesis that nevi are associated with breast tumor risk, we explored the relationships between number of nevi and benign and malignant breast disease risk.

**Methods and Findings:**

We prospectively analyzed data from E3N, a cohort of French women aged 40–65 y at inclusion in 1990. Number of nevi was collected at inclusion. Hazard ratios (HRs) for breast cancer and 95% confidence intervals (CIs) were calculated using Cox proportional hazards regression models. Associations of number of nevi with personal history of benign breast disease (BBD) and family history of breast cancer were estimated using logistic regression. Over the period 15 June 1990–15 June 2008, 5,956 incident breast cancer cases (including 5,245 invasive tumors) were ascertained among 89,902 women. In models adjusted for age, education, and known breast cancer risk factors, women with “very many” nevi had a significantly higher breast cancer risk (HR = 1.13, 95% CI = 1.01–1.27 versus “none”; *p*_trend_ = 0.04), although significance was lost after adjustment for personal history of BBD or family history of breast cancer. The 10-y absolute risk of invasive breast cancer increased from 3,749 per 100,000 women without nevi to 4,124 (95% CI = 3,674–4,649) per 100,000 women with “very many” nevi. The association was restricted to premenopausal women (HR = 1.40, *p*_trend_ = 0.01), even after full adjustment (HR = 1.34, *p*_trend_ = 0.03; *p*_homogeneity_ = 0.04), but did not differ according to breast cancer type or hormone receptor status. In addition, we observed significantly positive dose–response relationships between number of nevi and history of biopsy-confirmed BBD (*n = *5,169; *p*_trend_<0.0001) and family history of breast cancer in first-degree relatives (*n = *7,472; *p*_trend_ = 0.0003). The main limitations of our study include self-report of number of nevi using a qualitative scale, and self-reported history of biopsied BBD.

**Conclusions:**

Our findings suggest associations between number of nevi and the risk of premenopausal breast cancer, BBD, and family history of breast cancer. More research is warranted to elucidate these relationships and to understand their underlying mechanisms.

*Please see later in the article for the Editors' Summary*

## Abstract 2

Using data from the French E3N prospective cohort, Marina Kvaskoff and colleagues examine the association between number of cutaneous nevi and the risk for breast cancer.

*Please see later in the article for the Editors' Summary*

## Editors' Summary

**Background:**

In 2012, nearly 1.7 million women worldwide discovered they had breast cancer, and about half a million women died from the disease. Breast cancer begins when cells in the breast acquire genetic changes that allow them to divide uncontrollably and to move around the body (metastasize). Uncontrolled cell division leads to the formation of a lump that can be detected by mammography (a breast X-ray) or by manual breast examination. Breast cancer is treated by surgical removal of the lump, or, if the cancer has started to spread, by removal of the whole breast (mastectomy). Surgery is usually followed by radiotherapy or chemotherapy to kill any remaining cancer cells. Because the female sex hormones estrogen and progesterone stimulate the growth of some tumors, drugs that block hormone receptors are also used to treat receptor-positive breast cancer. Nowadays, the prognosis (outlook) for women with breast cancer is good, and in developed countries, nearly 90% of affected women are still alive five years after diagnosis.

**Why Was This Study Done?:**

Several hormone-related factors affect a woman's chances of developing breast cancer. For example, women who have no children or who have them late in life have a higher breast cancer risk than women who have several children when they are young because pregnancy alters sex hormone levels. Interestingly, the development of moles (nevi)—dark skin blemishes that are risk factors for the development of melanoma, a type of skin cancer—may also be affected by estrogen and progesterone. Thus, the number of nevi might be a marker of blood hormone levels and might predict breast cancer risk. In this prospective cohort study, the researchers test this hypothesis by investigating the association between how many moles a woman has and her breast cancer risk. A prospective cohort study enrolls a group (cohort) of people, determines their baseline characteristics, and follows them over time to see which characteristics are associated with the development of specific diseases.

**What Did the Researchers Do and Find?:**

In 1990, the E3N prospective cohort study enrolled nearly 100,000 French women (mainly school teachers) aged 40–65 years to investigate cancer risk factors. The women completed a baseline questionnaire about their lifestyle and medical history, and regular follow-up questionnaires that asked about cancer occurrence. In the initial questionnaire, the women indicated whether they had no, a few, many, or very many moles. Between 1990 and 2008, nearly 6,000 women in the cohort developed breast cancer. Using statistical methods to calculate hazard ratios (an “HR” compares how often a particular event happens in two groups with different characteristics; an HR greater than one indicates that a specific characteristic is associated with an increased risk of the event), the researchers report that women with “very many” nevi had a significantly higher breast cancer risk (a higher risk that was unlikely to have occurred by chance) than women with no nevi. Specifically, the age-adjusted HR for breast cancer among women with “very many” nevi compared to women with no nevi was 1.17. After adjustment for a personal history of benign (noncancerous) breast disease and a family history of breast cancer (two established risk factors for breast cancer), the association between nevi and breast cancer risk among the whole cohort became nonsignificant. Notably, however, the association among only premenopausal women remained significant after full adjustment (HR = 1.34), which corresponded to an increase in ten-year absolute risk of invasive breast cancer from 2,515 per 100,000 women with no nevi to 3,370 per 100,000 women with “very many” nevi.

**What Do These Findings Mean?:**

These findings suggest that among premenopausal women there is a modest association between nevi number and breast cancer risk. This noncausal relationship may indicate that nevi and breast diseases are affected in similar ways by hormones or share common genetic factors, but the accuracy of these findings may be limited by aspects of the study design. For example, self-report of nevi numbers using a qualitative scale may have introduced some inaccuracies into the estimates of the association between nevi number and breast cancer risk. Most importantly, these findings are insufficient to support the use of nevi counts in breast cancer screening or diagnosis. Rather, together with the findings reported by Zhang et al. in an independent *PLOS Medicine* Research Article, they suggest that further studies into the biological mechanisms underlying the relationship between nevi and breast cancer and the association itself should be undertaken.

**Additional Information:**

Please access these websites via the online version of this summary at http://dx.doi.org/10.1371/journal.pmed.1001660.

- This study is further discussed in a *PLOS Medicine* Perspective by Fuhrman and Cardenas
- An independent *PLOS Medicine* Research Article by Zhang et al. also investigates the relationship between nevi number and breast cancer risk
- The US National Cancer Institute provides comprehensive information about cancer (in English and Spanish), including detailed information for patients and professionals about breast cancer; it also has a fact sheet on moles
- Cancer Research UK, a not-for profit organization, provides information about cancer, including detailed information on breast cancer
- The UK National Health Service Choices website has information and personal stories about breast cancer; the not-for profit organization Healthtalkonline also provides personal stories about dealing with breast cancer
- More information about the E3N prospective cohort study is available; detailed information is available in French

## Introduction

Melanocytic nevi (hereafter referred to as nevi) are benign skin tumors resulting from epidermal melanocyte proliferation. They occur more frequently in fair- than in dark-skinned individuals and can be either congenital or acquired later in life. Nevus acquisition starts early in childhood and peaks during puberty, then declines in adulthood, with progressive loss with age [1]. Twin studies have consistently demonstrated genetic heritability of number of nevi [2]–[4], with an estimated 40%–80% of the variance in nevus counts being attributable to genetic effects [2],[4]. Genome-wide association studies have indeed reported several genes involved in nevus count, including *CDKN2A* and *MTAP*, both located at the 9p21 locus, and *PLA2G6*, located at 22q13 [5],[6]. Childhood sun exposure is also likely to play an important role in nevus acquisition, as suggested by studies of site distribution of nevi in children, which have consistently shown higher nevus densities on habitually sun-exposed body sites [7]–[10]. Number of sunburns [8],[11]–[13] and low latitude [12],[14]–[18] have also been associated with increased nevus prevalence. Because number of nevi peaks during puberty [1] and nevi are reported to be darker and larger during pregnancy [19],[20], a hormonal influence on nevi may also be speculated. Consistently, melanocytes, the pigment-producing cells, have been shown to express estrogen and androgen receptors [21]. However, the role of sex hormones in the occurrence of nevi remains to be determined.

Number of nevi is the strongest known risk factor for cutaneous melanoma [22], and an association has been suggested between melanoma and breast cancer [23],[24]. In addition, number of nevi and melanoma risk have been associated with variants in the *CDKN2A* gene [6], which plays a role in cell cycle regulation, while *CDKN2A* inactivation has been shown to be common in breast cancer [25]. Number of nevi has also been found to be associated with several benign and malignant diseases, particularly hormone-related conditions, including endometriosis [26], leiomyoma [27], and thyroid diseases [28]. Whether these associations are explained by common hormonal or genetic pathways has not yet been clarified.

In order to investigate whether nevus count is associated with breast tumor risk, we explored the relationships between number of nevi and the risks of benign and malignant breast diseases and family history of breast cancer, in the French E3N prospective cohort.

## Methods

### Ethics Statement

The E3N cohort received ethical approval from the French National Commission for Computed Data and Individual Freedom (Commission Nationale de l'Informatique et des Libertés), and all participants in the study provided informed consent.

### The E3N Cohort

E3N is a prospective cohort study involving 98,995 women born in 1925–1950, living in metropolitan France at inclusion and insured by the Mutuelle Générale de l'Éducation Nationale, a national health scheme primarily covering teachers. The cohort has been described in detail elsewhere [29]. Briefly, women were enrolled from February 1, 1989, through November 30, 1991, after returning a baseline self-administered questionnaire on their lifestyle and medical history. Follow-up questionnaires were sent every 2–3 y thereafter.

### Breast Cancer Assessment

All cohort questionnaires inquired about the occurrence of cancer, including breast cancer, requesting contact details of the participants' physicians and permission to contact them. A small number of breast cancer cases were further identified from insurance files and death certificates. Pathology reports were obtained for 93% of incident cases. We also considered cases for which pathology reports had not been obtained, because the proportion of false-positive self-reports was low in our study population (<5%). Information on ascertained estrogen receptor (ER) and progesterone receptor (PR) status was extracted from pathology reports, and invasive breast cancer cases were classified accordingly into four categories: ER+/PR+, ER+/PR−, ER−/PR+, and ER−/PR−. Women with unknown receptor status—mostly with tumors diagnosed in the early years of follow-up, when determining hormone receptor status was not compulsory (*n = *1,415, 27% of the tumors)—were excluded from the analyses stratified according to hormone receptor status.

### Benign Breast Disease Ascertainment

The 1990 and 1992 questionnaires asked women to report if they had ever had a personal history of benign breast disease (BBD) (breast adenoma or fibroadenoma, fibrocystic breast disease, or other) before inclusion in the cohort (prevalent BBD, *n = *19,742). Women also reported whether a biopsy had been performed for these diseases. This information was then collected prospectively in subsequent questionnaires (incident BBD, *n = *11,600). To avoid the potential selection bias of including only late-diagnosed BBD, both prevalent and incident BBDs were included in the analyses. When history of BBD was analyzed as a potential confounder, both biopsied and non-biopsied BBDs were included in order to maximize statistical power; however, we performed a sensitivity analysis excluding non-biopsied BBDs and verified the stability of the findings. When history of BBD was analyzed as an outcome, only biopsy-confirmed BBDs were considered in order to minimize misclassification.

### Assessment of Family History of Breast Cancer

Family history of breast cancer was collected in the inclusion questionnaire, where participants were asked to self-report whether their first-degree relatives had ever had a history of breast cancer.

### Assessment of Exposure and Covariates

The inclusion questionnaire asked women to self-report their number of moles, with four possible answers: none, a few, many, or very many. The questionnaire also inquired about education level, skin complexion, height and weight, and physical activity. Data on age at menarche, parity, age at first full-term pregnancy, and breastfeeding were collected in the inclusion (1990) and 1992 questionnaires. Data on use of oral contraceptives (OCs), premenopausal progestogens, and menopausal hormone therapy (MHT) were collected in 1992 and updated in each follow-up questionnaire. Body mass index (BMI) was calculated as weight in kilograms, reported at inclusion and updated at each follow-up questionnaire, divided by height in meters squared. Menopausal status, age at menopause, and history of recent mammographic exam (in the period since the previous questionnaire) were collected at inclusion and at each follow-up questionnaire. To obtain average levels of residential sun exposure during childhood and adulthood, we linked data on county of birth and county of residence at inclusion to a database from the Joint Research Centre of the European Commission containing mean daily ultraviolet (UV) radiation dose in French counties [30]. Physical activity, height, and UV dose in county of birth and of residence at baseline were analyzed in tertiles. BMI was grouped using World Health Organization cutoff points of 18.5 and 25 kg/m^2^, further dividing those with BMI 18.5–25 kg/m^2^ into the categories 18.5–22.5 and 22.5–25 kg/m^2^, according to the median BMI in our study population. Age at menarche, age at first full-term pregnancy, and age at menopause were categorized according to the median for these variables in the cohort. Women were considered postmenopausal if they reported 12 consecutive months of amenorrhea (unless due to hysterectomy), bilateral oophorectomy, or use of MHT, or if they self-reported being postmenopausal. Date of menopause was defined as the date of last menstrual period (unless due to hysterectomy, and if the last menstrual period occurred before MHT use), date of bilateral oophorectomy, or, in decreasing order of priority, self-reported date of menopause, date MHT use began, or date menopausal symptoms began; if no information on date of menopause was available, date of menopause was defined as the date corresponding to age 47 y if menopause was known to be artificial, or age 51 y otherwise, i.e., the median ages for artificial and natural menopause in the cohort, respectively.

### Statistical Analyses

Statistical analyses were performed using SAS software (version 9.3, SAS Institute). Hazard ratios (HRs) for breast cancer and 95% confidence intervals (CIs) were estimated using Cox proportional hazards regression models with age as the timescale. We also calculated absolute risks of breast cancer associated with the number of nevi. The association between number of nevi and breast cancer risk was assessed using different adjustment models. We first used age-adjusted models stratified by birth cohort in 5-y categories (Model 1) and then built a model including as covariates only those factors whose inclusion resulted in a 10% change of the HR for number of nevi. Besides age and birth cohort, this model included education (<12, 12–14, or ≥15 y), use of premenopausal progestogens (ever/never), menopausal status (pre- or postmenopausal), age at menopause in postmenopausal women (<51 or ≥51 y), and use of MHT in postmenopausal women (ever/never) (Model 2). Because further adjustment for personal history of BBD or family history of breast cancer resulted in the largest changes in HRs for breast cancer, we evaluated their effect separately by creating two models, Model 2 further adjusted for personal history of BBD (Model 3) and Model 3 additionally adjusted for family history of breast cancer (Model 4). Finally, a fully adjusted model included all the preceding covariates along with the remaining potential confounders, including BMI, height, physical activity, age at menarche, parity, age at first full-term pregnancy, breastfeeding, use of OCs, personal history of mammographic exam, and UV dose in county of birth and of residence at cohort inclusion (Model 5). All models were generated both for in situ and invasive breast cancer cases combined, and for in situ and invasive cases separately, and homogeneity tests were used to compare estimates between in situ and invasive cases. We performed tests for linear trend across categories of number of nevi by assigning a numerical value to each category. Effect modification was evaluated using interaction tests. In addition, we stratified the analyses according to menopausal status, and we performed stratified analyses according to hormone receptor status or tumor histological type in invasive cases using competing-risk models [16]. We performed homogeneity tests to compare risk estimates across strata.

We additionally used a case–control design to explore the relationships between number of nevi and (1) personal history of BBD and (2) family history of breast cancer in first-degree relatives. For this analysis, unconditional logistic regression models were used to estimate odds ratios (ORs) and 95% CIs. For history of BBD, potential confounders were included only if their inclusion resulted in at least a 10% change in the OR. Final models included age at cohort inclusion, age at last returned questionnaire, education, premenopausal progestogens, menopausal status, age at menopause, use of MHT in postmenopausal women, and family history of breast cancer. For family history of breast cancer, models included education and age at cohort inclusion.

For all analyses, missing values in covariates were imputed to the modal category if values were missing in <5% of observations, otherwise a “missing” category was created for the covariate. Regarding our exposure of interest, women with no information on number of nevi (*n = *2,211; 2.2% of the total cohort) were more likely to be older, to have a late menarche, to be younger at their first full-term pregnancy, and to be nulliparous, but were less likely to have a high education level or a high physical activity level at baseline; to be overweight or obese; to be tall; to have ever used OCs, premenopausal progestogens, or MHT; to have ever breastfed; to have ever had a history of BBD, a mammographic exam, or a family history of breast cancer; or to have a high UV dose in their county of residence at baseline compared to those with available data on this factor (Table S1). However, given the low rate of missing values in this variable (2.2%), we speculate that exclusion of missing data would have little impact on the findings; we thus excluded participants lacking information on number of nevi from all analyses.

### Population for Analysis

From the original study population, we excluded women with missing data on number of nevi (*n = *2,211), with a cancer history before inclusion (*n = *5,024), lost to follow-up after they replied to the inclusion questionnaire (*n = *1,931), or with primary amenorrhea (*n = *27). Our final sample for analysis consisted of 89,802 women. Woman-years were computed from the date the first questionnaire was returned to the date of diagnosis of breast cancer or any other cancer, date of last questionnaire returned, or date of end of follow-up (June 25, 2008), whichever occurred first.

For the analyses exploring number of nevi in relation to history of BBD, we further excluded women with non-biopsied BBD (*n = *26,173). For analyses exploring family history of breast cancer, we excluded from the original study population women with missing data on number of nevi (*n = *2,211) and those with missing data on family history of breast cancer (*n = *728). The final study populations for these analyses included 63,629 women for history of BBD and 96,056 women for family history of breast cancer.

## Results

Over 1,385,970 woman-years and a median follow-up of 17.9 y, a total of 5,956 incident breast cancer cases (including 5,245 invasive and 711 in situ cases) were ascertained among the 89,902 included women. Table 1 describes the baseline characteristics of the participants according to number of nevi. Women with a high number of nevi were more likely to be from more recent birth cohorts; to have a high education level; to be tall (≥164 cm); to have an early menarche (<13 y of age); to have ever used OCs, premenopausal progestogens, or MHT; to have ever breastfed; to be premenopausal; and to have a history of BBD. In contrast, they were less likely to be physically active, to be overweight or obese, to have had three or more full-term pregnancies, and to have had their first live birth before the age of 30 y.

**Table 1:** Baseline characteristics of the study population.

Characteristic	Number of Nevi				*p*-Value^a^
	None (*n = *9,044)	A Few (*n = *33,187)	Many (*n = *37,911)	Very Many (*n = *9,660)	
**Year of birth**					
<1930	1,266 (14.0)	2,913 (8.8)	2,143 (5.7)	382 (4.0)	<0.0001
1930–1934	1,684 (18.6)	4,790 (14.4)	4,028 (10.6)	785 (8.1)	
1935–1939	2,053 (22.7)	6,738 (20.3)	6,654 (17.5)	1,332 (13.8)	
1940–1945	1,911 (21.1)	7,891 (23.8)	9,538 (25.2)	2,389 (24.7)	
≥1950	2,130 (23.6)	10,855 (32.7)	15,548 (41.0)	4,772 (49.4)	
**Education**					
<12 y	1,724 (19.1)	1,724 (14.9)	4,519 (11.9)	804 (8.3)	<0.0001
12–14 y	4,766 (52.7)	4,766 (52.6)	19,466 (51.4)	4,792 (49.6)	
≥15 y	2,554 (28.2)	2,554 (32.5)	13,926 (36.7)	4,064 (42.1)	
**Physical activity at inclusion (MET-h/wk)**					
<13.8	2,274 (25.1)	7,820 (23.6)	8,814 (23.3)	2,328 (24.1)	<0.0001
13.8–25.0	3,147 (34.8)	12,193 (36.7)	14,448 (38.1)	3,831 (39.7)	
≥25.0	3,623 (40.1)	13,174 (39.7)	14,649 (38.6)	3,501 (36.2)	
**BMI (kg/m^2^)**					
<18.5	343 (3.8)	1,336 (4.0)	1,740 (4.6)	496 (5.1)	<0.0001
18.5–22.4	4,378 (48.4)	17,674 (53.3)	20,724 (54.7)	5,446 (56.4)	
22.5–24	2,448 (27.1)	8,332 (25.1)	9,252 (24.4)	2,254 (23.3)	
≥25	1,875 (20.7)	5,845 (17.6)	6,195 (16.3)	1,464 (15.2)	
**Height (cm)**					
<160	3,299 (36.5)	11,285 (34.0)	11,968 (31.6)	2,758 (28.5)	<0.0001
160–163	2,728 (30.2)	10,068 (30.3)	11,383 (30.0)	2,933 (30.4)	
≥164	3,017 (33.3)	11,834 (35.7)	14,560 (38.4)	3,969 (41.1)	
**Age at menarche**					
<13 y	3,876 (42.9)	14,916 (45.0)	17,377 (45.8)	4,485 (46.4)	<0.0001
≥13 y	5,168 (57.1)	18,271 (55.0)	20,534 (54.2)	5,175 (53.6)	
**OC use**					
Never	4,515 (49.9)	13,979 (42.1)	13,873 (36.6)	3,052 (31.6)	<0.0001
Ever	4,529 (50.1)	19,208 (57.9)	24,038 (63.4)	6,608 (68.4)	
**Age at first full-term pregnancy (in parous women)**					
<30 y	7,025 (89.1)	26,091 (88.7)	29,431 (87.5)	7,298 (85.7)	<0.0001
≥30 y	857 (10.9)	3,336 (11.3)	4,221 (12.5)	1,221 (14.3)	
**Parity**					
Nulliparous	1,162 (12.9)	3,762 (11.3)	4,259 (11.2)	1,141 (11.8)	<0.0001
1–2 full-term pregnancies	5,020 (55.5)	19,515 (58.8)	22,806 (60.2)	5,923 (61.3)	
≥3 full-term pregnancies	2,862 (31.6)	9,910 (29.9)	10,846 (28.6)	2,596 (26.9)	
**Breastfeeding** b					
Never	3,462 (38.3)	12,353 (37.2)	13,540 (35.7)	3,411 (35.3)	<0.0001
Ever	4,533 (50.1)	17,245 (52.0)	20,324 (53.6)	5,316 (55.0)	
Unknown	1,049 (11.6)	3,589 (10.8)	4,047 (10.7)	933 (9.7)	
**Use of premenopausal progestogens**					
Never	6,436 (71.2)	21,239 (64.0)	22,211 (58.6)	5,143 (53.2)	<0.0001
Ever	2,608 (28.8)	11,948 (36.0)	15,700 (41.4)	4,517 (46.8)	
**Menopausal status**					
Premenopausal	4,104 (45.4)	18,567 (56.0)	24,600 (64.9)	6,926 (71.7)	<0.0001
Postmenopausal	4,940 (54.6)	14,620 (44.0)	13,311 (35.1)	2,734 (28.3)	
**Age at menopause** c					
<51 y	2,907 (58.9)	8,707 (59.6)	8,172 (61.4)	1,732 (63.4)	<0.0001
≥51 y	2,033 (41.2)	5,913 (40.4)	5,139 (38.6)	1,002 (36.7)	
**MHT use** c					
Never	2,957 (59.9)	8,157 (55.8)	6,996 (52.6)	1,334 (48.8)	<0.0001
Ever	1,983 (40.1)	6,463 (44.2)	6,315 (47.4)	1,400 (51.2)	
**History of BBD**					
Never	7,410 (81.9)	26,240 (79.1)	29,309 (77.3)	7,101 (73.5)	<0.0001
Ever	1,634 (18.1)	6,947 (20.9)	8,602 (22.7)	2,559 (26.5)	
**History of mammographic exam**					
Never	2,746 (30.4)	9,662 (29.1)	10,824 (28.6)	2,733 (28.3)	0.003
Ever	6,298 (69.6)	23,525 (70.9)	27,087 (71.5)	6,927 (71.7)	
**Family history of breast cancer**					
No	8,414 (93.0)	30,642 (92.3)	35,071 (92.5)	8,891 (92.0)	0.05
Yes	630 (7.0)	2,545 (7.7)	2,840 (7.5)	769 (8.0)	
**UV dose in county of birth** b					
<1.40	2,727 (30.2)	9,567 (28.8)	11,188 (29.5)	2,777 (28.8)	<0.0001
1.40–1.61	2,634 (29.1)	9,972 (30.1)	11,868 (31.3)	3,006 (31.1)	
≥1.61	2,994 (33.1)	10,982 (33.1)	11,699 (30.9)	2,938 (30.4)	
Missing	689 (7.6)	2,666 (8.0)	3,156 (8.3)	939 (9.7)	
**UV dose in county of residence at inclusion**					
<1.40	2,680 (29.6)	9,688 (29.2)	11,313 (29.8)	2,842 (29.4)	<0.0001
1.40–1.63	3,127 (34.6)	11,779 (35.5)	13,834 (36.5)	3,598 (37.3)	
≥1.63	3,237 (35.8)	11,720 (35.3)	12,764 (33.7)	3,220 (33.3)	

a From chi-square tests.

b Tests were performed excluding participants in the unknown/missing category.

c Among postmenopausal women.

MET, metabolic equivalent of task.

In age-adjusted models, women in the highest category of number of nevi had a significantly higher breast cancer risk (HR = 1.17, 95% CI = 1.05–1.31; ptrend = 0.006) compared to those in the lowest (Table 2). The association remained in Model 2; however, associations were reduced and were no longer statistically significant after additional adjustment for history of BBD (Model 3, HR = 1.10, 95% CI = 0.98–1.23) and family history of breast cancer (Model 4, HR = 1.09, 95% CI = 0.98–1.22). The association was observed for both in situ and invasive tumors, with no evidence of heterogeneity across these groups (*p*_homogeneity_ = 0.27 in Model 2); however, the HR for number of nevi in invasive cases was statistically significant in the model adjusted only for age (Model 1). The 10-y absolute risk of invasive breast cancer increased from 3,749 per 100,000 women without nevi to 4,124 (95% CI = 3,674–4,649) per 100,000 women with “very many” nevi.

**Table 2:** Hazard ratios and 95% confidence intervals for risk of breast cancer in relation to number of nevi, E3N cohort (*n = *89,802).

Number of Nevi	*n*	Cases	Model 1: Age-Adjusted HR (95% CI)	Model 2: Multivariable HR^a^ (95% CI)	Model 3: Multivariable HR^b^ (95% CI)	Model 4: Multivariable HR^c^ (95% CI)	Model 5: Multivariable HR^d^ (95% CI)
**All breast cancers**							
None	9,044	579	1.00 (Reference)	1.00 (Reference)	1.00 (Reference)	1.00 (Reference)	1.00 (Reference)
A few	33,187	2,194	1.04 (0.95–1.14)	1.02 (0.93–1.12)	1.01 (0.93–1.11)	1.01 (0.92–1.11)	1.01 (0.92–1.11)
Many	37,911	2,502	1.06 (0.97–1.16)	1.04 (0.95–1.14)	1.02 (0.93–1.12)	1.02 (0.93–1.11)	1.01 (0.92–1.11)
Very many	9,660	681	1.17 (1.05–1.31)	1.13 (1.01–1.27)	1.10 (0.98–1.23)	1.09 (0.98–1.22)	1.08 (0.96–1.21)
*p*_trend_			0.006	0.04	0.12	0.15	0.26
**In situ breast cancers**							
None	9,044	61	1.00 (Reference)	1.00 (Reference)	1.00 (Reference)	1.00 (Reference)	1.00 (Reference)
A few	33,187	258	1.13 (0.86–1.50)	1.11 (0.84–1.46)	1.10 (0.93–1.45)	1.09 (0.82–1.44)	1.09 (0.92–1.44)
Many	37,911	297	1.15 (0.87–1.52)	1.11 (0.84–1.46)	1.09 (0.93–1.44)	1.08 (0.82–1.43)	1.08 (0.82–1.43)
Very many	9,660	95	1.47 (1.06–2.04)	1.39 (1.01–1.93)	1.35 (0.98–1.87)	1.34 (0.97–1.85)	1.33 (0.96–1.84)
*p*_trend_			0.03	0.08	0.12	0.13	0.15
**Invasive breast cancers**							
None	9,044	518	1.00 (Reference)	1.00 (Reference)	1.00 (Reference)	1.00 (Reference)	1.00 (Reference)
A few	33,187	1,936	1.03 (0.93–1.13)	1.01 (0.92–1.12)	1.00 (0.91–1.11)	1.00 (0.91–1.10)	1.00 (0.91–1.10)
Many	37,911	2,205	1.05 (0.95–1.16)	1.03 (0.93–1.13)	1.01 (0.92–1.12)	1.01 (0.92–1.11)	1.00 (0.91–1.10)
Very many	9,660	586	1.13 (1.01–1.28)	1.10 (0.98–1.24)	1.07 (0.95–1.21)	1.06 (0.94–1.20)	1.05 (0.93–1.18)
*p*_trend_			0.03	0.11	0.27	0.32	0.50

a Adjusted for age (timescale), education, menopausal status, age at menopause (in postmenopausal women), use of MHT (in postmenopausal women), and use of premenopausal progestogens, and stratified according to year of birth in 5-y categories.

b Model 2 additionally adjusted for personal history of BBD.

c Model 3 additionally adjusted for personal history of BBD and family history of breast cancer.

d Model 4 additionally adjusted for BMI, height, physical activity, age at menarche, age at first full-term pregnancy, parity, breastfeeding, use of OCs, history of mammographic exam, UV dose in county of birth, and UV dose in county of residence at inclusion.

Although “none” was the obvious reference category to explore number of nevi, it was the smallest category in our cohort, and it could be argued that this could inflate error estimates. However, since the “none” group was not small per se—it included over 9,000 participants—and since our findings were not substantially modified when testing different reference groups for this variable (“none/a few”: Table S2; “a few”: Table S3), we kept “none” as the reference category for number of nevi in all subsequent analyses.

No significant interactions were found between number of nevi and history of BBD (*p*_interaction_ = 0.27) or family history of breast cancer (*p*_interaction_ = 0.97) for the risk of breast cancer. We also detected no significant interaction of number of nevi with skin color (*p*_interaction_ = 0.73), physical activity at inclusion (*p*_interaction_ = 0.17), UV dose in county of birth (*p*_interaction_ = 0.07), or UV dose in county of residence at inclusion (*p*_interaction_ = 0.06). Since the *p*-values for the last two factors were close to statistical significance, we conducted stratified analyses according to the median of UV dose in county of birth (Table S4) and according to the median of UV dose in county of residence at baseline (Table S5); however, we observed no significant heterogeneity across these strata.

When stratifying the analysis according to menopausal status, we detected significant heterogeneity across strata (*p*_homogeneity_ = 0.04). The association was restricted to premenopausal women, in whom the relation remained even in the multivariable model (HR = 1.34, 95% CI = 1.00–1.81 for “very many” versus “none”; *p*_trend_ = 0.03) (Table 3), corresponding to a 10-y absolute risk of invasive breast cancer of 2,515 per 100,000 women with no nevi versus 3,370 (95% CI = 2,515–4,552) per 100,000 women with “very many” nevi. Subgroup analyses according to histological type of breast cancer (ductal carcinoma, lobular carcinoma, or other) (Table 4) or ER/PR status (Table 5) yielded no significant heterogeneity.

**Table 3:** Hazard ratios and 95% confidence intervals for risk of breast cancer in relation to number of nevi, stratified by menopausal status, E3N cohort (*n = *89,802).

Number of Nevi	Premenopausal			Postmenopausal			*p*_homogeneity_
	Cases	Multivariable HR^a^ (95% CI)	Multivariable HR^b^ (95% CI)	Cases	Multivariable HR^a^ (95% CI)	Multivariable HR^b^ (95% CI)	
None	59	1.00 (Reference)	1.00 (Reference)	459	1.00 (Reference)	1.00 (Reference)	
A few	329	1.15 (0.87–1.52)	1.14 (0.86–1.50)	1,607	1.00 (0.90–1.11)	0.98 (0.88–1.09)	
Many	491	1.25 (0.95–1.63)	1.22 (0.93–1.60)	1,714	0.99 (0.89–1.10)	0.97 (0.87–1.08)	
Very many	160	1.40 (1.04–1.89)	1.34 (1.00–1.81)	426	1.03 (0.90–1.18)	0.99 (0.87–1.14)	
*p*_trend_		0.01	0.03		0.79	0.82	0.04

a Adjusted for age (timescale), education, age at menopause (in postmenopausal women), use of MHT (in postmenopausal women), and use of premenopausal progestogens, and stratified according to year of birth in 5-y categories (Model 2).

b Additionally adjusted for personal history of BBD and family history of breast cancer (model used for homogeneity test) (Model 4).

**Table 4:** Hazard ratios and 95% confidence intervals for risk of breast cancer in relation to number of nevi, stratified by histological type of breast cancer, E3N cohort (*n* = 89,429).

Number of Nevi	Ductal Carcinoma			Lobular Carcinoma			Other Types			*p*_homogeneity_
	Cases	Multivariable HR^a^ (95% CI)	Multivariable HR^b^ (95% CI)	Cases	Multivariable HR^a^ (95% CI)	Multivariable HR^b^ (95% CI)	Cases	Multivariable HR^a^ (95% CI)	Multivariable HR^b^ (95% CI)	
None	332	1.00 (Reference)	1.00 (Reference)	89	1.00 (Reference)	1.00 (Reference)	52	1.00 (Reference)	1.00 (Reference)	
A few	1,280	1.04 (0.92–1.17)	1.03 (0.91–1.16)	291	0.89 (0.70–1.13)	0.88 (0.69–1.11)	226	1.17 (0.86–1.58)	1.15 (0.85–1.55)	
Many	1,472	1.06 (0.94–1.20)	1.04 (0.92–1.17)	331	0.90 (0.71–1.14)	0.89 (0.70–1.12)	251	1.15 (0.85–1.55)	1.12 (0.83–1.51)	
Very many	391	1.13 (0.98–1.32)	1.09 (0.94–1.27)	92	1.01 (0.75–1.35)	0.97 (0.72–1.31)	65	1.19 (0.82–1.72)	1.14 (0.78–1.64)	
*p*_trend_		0.09	0.24		0.89	0.95		0.52	0.69	0.85

a Adjusted for age (timescale), education, menopausal status, age at menopause (in postmenopausal women), use of MHT (in postmenopausal women), and use of premenopausal progestogens, and stratified according to year of birth in 5-y categories (Model 2).

b Additionally adjusted for personal history of BBD and family history of breast cancer (model used for homogeneity test) (Model 4).

**Table 5:** Hazard ratios and 95% confidence intervals for risk of breast cancer in relation to number of nevi, stratified by hormonal receptor status, E3N cohort (*n = *88,387).

Number of Nevi	ER+/PR+			ER+/PR−			ER−/PR+			ER−/PR−			*p*_homogeneity_
	Cases	Multivariable HR^b^ (95% CI)	Multivariable HR^c^ (95% CI)	Cases	Multivariable HR^b^ (95% CI)	Multivariable HR^c^ (95% CI)	Cases	Multivariable HR^b^ (95% CI)	Multivariable HR^c^ (95% CI)	Cases	Multivariable HR^b^ (95% CI)	Multivariable HR^c^ (95% CI)	
None	226	1.00 (Reference)	1.00 (Reference)	68	1.00 (Reference)	1.00 (Reference)	8	1.00 (Reference)	1.00 (Reference)	72	1.00 (Reference)	1.00 (Reference)	
A few	834	1.00 (0.86–1.16)	0.99 (0.85–1.14)	285	1.14 (0.88–1.49)	1.12 (0.86–1.46)	71	2.22 (1.07–4.61)	2.19 (1.05–4.55)	225	0.84 (0.64–1.09)	0.82 (0.63–1.08)	
Many	953	1.02 (0.88–1.18)	1.00 (0.86–1.16)	331	1.19 (0.92–1.55)	1.17 (0.90–1.52)	64	1.66 (0.80–3.48)	1.64 (0.79–3.44)	265	0.87 (0.67–1.13)	0.85 (0.66–1.10)	
Very many	251	1.09 (0.90–1.30)	1.05 (0.87–1.26)	80	1.17 (0.84–1.62)	1.12 (0.81–1.55)	18	1.78 (0.77–4.12)	1.73 (0.75–4.00)	79	1.04 (0.75–1.44)	1.00 (0.72–1.38)	
*p*_trend_		0.34	0.57		0.30	0.44		0.99	0.93		0.65	0.81	0.96

Women with missing information on hormone receptor status were excluded from this analysis (*n = *1,415).

b Adjusted for age (timescale), education, menopausal status, age at menopause (in postmenopausal women), use of MHT (in postmenopausal women), and use of premenopausal progestogens, and stratified according to year of birth in 5-y categories (Model 2).

c Additionally adjusted for personal history of BBD and family history of breast cancer (model used for homogeneity test) (Model 4).

Associations between number of nevi and BBD and family history of breast cancer are reported in Tables 6 and 7. We observed positive dose–response relationships between number of nevi and a personal history of biopsy-confirmed BBD (ORs of 1.14, 1.15, and 1.26 across increasing categories of number of nevi; *p*_trend_<0.0001) and family history of breast cancer in first-degree relatives (ORs of 1.14, 1.15, and 1.25 across increasing categories of number of nevi; *p*_trend_ = 0.0003).

**Table 6:** Odds ratios and 95% confidence intervals for number of nevi in relation to history of benign breast disease, E3N cohort.

Number of Nevi	*n*	History of BBD	Crude OR (95% CI)	Multivariable OR^a^ (95% CI)
None	6,852	427	1.00 (Reference)	1.00 (Reference)
A few	23,930	1,813	1.23 (1.11–1.38)	1.14 (1.01–1.29)
Many	26,417	2,251	1.40 (1.26–1.56)	1.15 (1.02–1.30)
Very many	6,430	678	1.77 (1.56–2.01)	1.26 (1.08–1.47)
*p*_trend_			<0.0001	0.008

a Adjusted for age at cohort inclusion, age at last returned questionnaire, education, menopausal status, age at menopause (in postmenopausal women), use of MHT (in postmenopausal women), use of premenopausal progestogens, and family history of breast cancer.

**Table 7:** Odds ratios and 95% confidence intervals for number of nevi in relation to family history of breast cancer, E3N cohort.

Number of Nevi	*n*	Family History of Breast Cancer	Crude OR (95% CI)	Multivariable OR^a^ (95% CI)
None	9,657	693	1.00 (Reference)	1.00 (Reference)
A few	35,503	2,791	1.10 (1.01–1.20)	1.14 (1.05–1.24)
Many	40,475	3,130	1.08 (1.00–1.18)	1.15 (1.06–1.25)
Very many	10,421	858	1.16 (1.05–1.29)	1.25 (1.13–1.39)
*p*_trend_			0.05	0.0003

a Adjusted for education and age at inclusion.

## Discussion

In the present study, we found a modest association between number of nevi and overall breast cancer risk, which was restricted to premenopausal women. In these women, the highest versus lowest category of number of nevi was associated with a HR of 1.34 for breast cancer (10-y absolute risk of invasive breast cancer of 2,515 per 100,000 women with no nevi versus 3,370 [95% CI = 2,515–4,552] per 100,000 women with “very many” nevi). A high number of nevi was also associated with the risk of biopsy-confirmed BBD and with family history of breast cancer in first-degree relatives. While adjustment for personal history of BBD and family history of breast cancer reduced the association between number of nevi and overall breast cancer risk, it did not substantially modify the association with premenopausal breast cancer risk.

While a causal relationship between number of nevi and breast disease risk seems unlikely, our observed associations between number of nevi and risk of premenopausal breast cancer, history of BBD, and family history of breast cancer may suggest at least two potential mechanisms.

One potential mechanism is that these relations could reflect a common hormonal influence on nevi and breast diseases. The fact that significant associations were restricted to premenopausal breast cancer and that number of nevi was associated with both BBD and breast cancer risk are consistent with this hypothesis, although we failed to find a stronger association with ER+ than with ER− tumors, possibly because of a lack of power in subgroup analyses. Consistent with this hypothesis, melanocytes are known to express estrogen and androgen receptors [21], melanogenesis is known to be influenced by endogenous sex hormones [21],[31], and a transient increase in nevi darkness and diameter has been observed during pregnancy [19],[20],[32]. Nonetheless, if this hypothesis were true, then an increase in nevus number should also be observed during pregnancy or MHT use, when endogenous female hormone levels are substantially increased. However, no previous study to our knowledge has made this observation to date.

A second potential mechanism is that nevi and breast diseases could share genetic factors, which is consistent with our observed association between number of nevi and family history of breast cancer. Interestingly, several studies have reported associations between melanoma and breast cancer, finding either a higher risk of breast cancer following melanoma diagnosis [33]–[37] or the opposite [23],[24],[34],[38]–[48], and a higher breast cancer risk was reported in melanoma-prone families carrying *CDKN2A* mutations [49]. Among genetic factors that could account for a common heritability between nevus count and breast cancer, one potential candidate is *CDKN2A*, a tumor suppressor gene encoding cyclin-dependent kinase inhibitors known to be frequently mutated or suppressed in a number of cancers. This gene has been identified as a high penetrance susceptibility gene for melanoma, with germline mutations occurring in 20%–40% of families with three or more melanoma cases [50], and it has also been associated with nevus count in recent genome-wide association studies [6],[51]. In addition, a SNP located in a block encompassing *CDKN2A* and *CDKN2B* at 9p21, rs1011970, was reported to be associated with breast cancer in a recent genome-wide scan [52]. The association was later confirmed in a pooled study, in which similar associations were reported in ER+ and ER− tumors [53].

*CDKN2A* codes for two proteins, p14 and p16 [54]. By competing with cyclin D1 for CDK4/6 binding, p16 inhibits the expression and transcription of cyclin D1, one of the main mediators of the proliferative action of estrogens [55]. Silencing of p16 protein expression through epigenetic mechanisms, or because of a germline mutation, has been suspected to play a crucial role in the progression of intraductal proliferative lesions [56] and has been associated with breast cancer risk, particularly in young women [57]. Moreover, estradiol-induced cell proliferation in the case of p16-enhanced cyclin D1 expression may be amplified in a highly estrogenic environment. This may be consistent with our finding that the association between number of nevi and breast cancer risk is restricted to premenopausal women.

However, because it is unclear whether the associations we found reflect common hormonal, genetic, or environmental pathways, more research is warranted to understand their underlying biological mechanisms.

Strengths of our study include the large sample size and prospective design of the E3N cohort; we also had detailed data on breast cancer cases, personal history of BBD, and family history of breast cancer.

The main limitation regarded self-report of nevi number, and use of a qualitative scale instead of counts. Repeatability studies of number of nevi indeed show a moderate reliability [58]–[60]. However, in this cohort of mostly educated women, self-reported features have demonstrated high reproducibility in several validation studies [61]–[63]. In addition, number of nevi showed a strong dose–response relationship with the risk of cutaneous melanoma in our cohort [64], which suggests satisfactory validity for this variable. Also, misclassification, if any, would be non-differential and independent from the studied outcomes, and would thus likely result in underestimating existing relationships.

Another limitation is that race information was not available in our cohort, as this type of question is not acceptable to French ethical committees. However, while E3N women did report their skin color, we detected no significant interaction in our findings according to this factor. Further, while the large sample size of our cohort makes it possible to detect small associations, our findings regarding breast cancer risk were of modest effect size and sensitive to adjustment, particularly for history of BBD and family history of breast cancer, for which we found an independent association with number of nevi. Also, given the number of interaction tests that we performed, some of our findings could be attributable to chance and should therefore be interpreted with caution. However, while a multiple testing issue can arise when a high number of hypothesis-free tests are performed [65], the significant interaction between menopausal status and nevi number with regard to breast cancer risk is based on known heterogeneity of breast cancer risk factors according to this characteristic.

Another limitation was that history of BBD was self-reported and could not always be confirmed through biopsy reports, which may have introduced some degree of misclassification. However, when we studied BBD as a potential confounder, our results were unchanged whether analyses included all BBDs or biopsied BBDs only, and when we studied history of BBD as an outcome, restricting the analyses to biopsy-confirmed BBD likely reduced this bias.

Another potential bias could arise from a complex relationship between UV exposure, number of nevi, and vitamin D levels. Indeed, exposure to UVB rays results in higher vitamin D synthesis, and normal versus insufficient or deficient vitamin D levels in adulthood have been associated with a reduced breast cancer risk [66]. Because number of nevi increases with sun exposure, UV exposure may act as a confounder in the association between number of nevi and breast cancer risk. Although our models were adjusted for residential UV dose, level of recreational sun exposure was not available, and we thus cannot rule out residual confounding, which would most likely result in reducing the strength of an association between number of nevi and breast cancer risk. Thus, it is unlikely that the observed associations between premenopausal breast cancer risk result from confounding, but regarding the absence of an association with postmenopausal breast cancer, we cannot rule out some residual negative confounding.

In conclusion, these data from a large prospective cohort study suggest associations between number of nevi and risk of premenopausal breast cancer, history of BBD, and family history of breast cancer. Because associations were modest and the results for breast cancer were sensitive to adjustment, we mostly consider our findings in terms of enhancement of our knowledge of pathophysiological mechanisms. More research is warranted before these findings could possibly be used in diagnosis or screening scores for breast cancer. If confirmed, these findings may suggest that nevi could be associated with other markers of breast cancer risk, such as mammographic density, which should warrant a specific study.

## Supporting Information

Table S1 **Baseline characteristics of the study population according to the availability of data on number of nevi.**
(DOCX)

Table S2 **Hazard ratios and 95% confidence intervals for number of nevi (in three categories, considering “none/a few” as the reference) in relation to the risk of breast cancer, E3N cohort (** ***n =*** **89,802).**
(DOCX)

Table S3 **Hazard ratios and 95% confidence intervals for number of nevi (in four categories, considering “a few” as the reference) in relation to the risk of breast cancer, E3N cohort (** ***n =*** **89,802).**
(DOCX)

Table S4 **Hazard ratios and 95% confidence intervals for number of nevi in relation to the risk of breast cancer, stratified by mean UV dose in county of birth, E3N cohort (** ***n =*** **89,802).**
(DOCX)

Table S5 **Hazard ratios and 95% confidence intervals for number of nevi in relation to the risk of breast cancer, stratified by mean UV dose in county of residence at baseline, E3N cohort (** ***n =*** **89,802).**
(DOCX)

## Abbreviations

BBD: benign breast disease
BMI: body mass index
CI: confidence interval
ER: estrogen receptor
HR: hazard ratio
MHT: menopausal hormone therapy
OC: oral contraceptive
OR: odds ratio
PR: progesterone receptor
UV: ultraviolet
