# Supplementary material for: Association between Melanocytic Nevi and Risk of Breast Diseases: The French E3N Prospective Cohort
Source: PLoS Med. 2014 Jun 10;11(6):e1001660. doi: 10.1371/journal.pmed.1001660 (PMC4051602; doi:10.1371/journal.pmed.1001660)
Supplement: Table S1 — Baseline characteristics of the study population according to the availability of data on number of nevi. (DOCX) [file pmed.1001660.s001.docx]

| **Table S1.** Baseline characteristics of the study population according to the availability of data on number of nevi | | | | | |
| --- | --- | --- | --- | --- | --- |
|  |  |  |  |  |  |
|  | **Data on number of nevi** | | | |  |
|  | **Missing**  **(n=2211)** | | **Non-missing**  **(n=96,784)** | | **P-value^a^** |
|  | **n** | **%** | **n** | **%** |  |
| **Year of birth** |  |  |  |  |  |
| <1930 | 442 | 20.0 | 13,407 | 13.9 | <0.0001 |
| 1930-1934 | 276 | 12.5 | 11,356 | 11.7 |  |
| 1935-1939 | 378 | 17.1 | 16,844 | 17.4 |  |
| 1940-1945 | 514 | 23.2 | 21,802 | 22.5 |  |
| ≥1950 | 601 | 27.2 | 33,375 | 34.5 |  |
| **Education** |  |  |  |  |  |
| <12 years | 206 | 9.3 | 12,023 | 12.4 | <0.0001 |
| 12-14 years | 1632 | 73.8 | 53,309 | 55.1 |  |
| ≥15 years | 373 | 16.9 | 31,452 | 32.5 |  |
| **Physical activity at inclusion (METs/h)** |  |  |  |  |  |
| <13.8 | 365 | 16.5 | 21,316 | 22.0 | <0.0001 |
| 13.8-25.0 | 1302 | 58.9 | 40,388 | 41.7 |  |
| ≥25.0 | 544 | 24.6 | 35,080 | 36.3 |  |
| **Body mass index (kg/m^2^)** |  |  |  |  |  |
| <18.5 | 41 | 1.8 | 3933 | 4.1 | <0.0001 |
| 18.5-22.4 | 1644 | 74.4 | 55,047 | 56.9 |  |
| 22.5-24 | 294 | 13.3 | 22,370 | 23.1 |  |
| ≥25 | 232 | 10.5 | 15,434 | 15.9 |  |
| **Height (cm)** |  |  |  |  |  |
| <160 | 688 | 31.1 | 29,420 | 30.4 | 0.01 |
| 160-163 | 823 | 37.2 | 33,850 | 35.0 |  |
| ≥164 | 700 | 31.7 | 33,514 | 34.6 |  |
| **Age at menarche** |  |  |  |  |  |
| <13 years | 699 | 31.6 | 40,774 | 42.1 | <0.0001 |
| ≥13 years | 1512 | 68.4 | 56,010 | 57.9 |  |
|  |  |  |  |  |  |
| **Oral contraceptive use** |  |  |  |  |  |
| Never | 1095 | 56.3 | 35,579 | 39.5 | <0.0001 |
| Ever | 850 | 43.7 | 54,554 | 60.5 |  |
| **Age at first full-term pregnancy (in parous women)** |  |  |  |  |  |
| <30 years | 1552 | 90.5 | 76,726 | 88.8 | 0.02 |
| ≥30 years | 162 | 9.5 | 9667 | 11.2 |  |
| **Parity** |  |  |  |  |  |
| Nulliparous | 497 | 22.5 | 10,391 | 10.7 | <0.0001 |
| 1-2 full-term pregnancies | 1210 | 54.7 | 60,089 | 62.1 |  |
| ≥3 full-term pregnancies | 504 | 22.8 | 26,304 | 27.1 |  |
| **Breastfeeding**^b^ |  |  |  |  |  |
| Never | 678 | 30.7 | 32,906 | 34.0 | 0.02 |
| Ever | 866 | 39.2 | 47,583 | 49.2 |  |
| Unknown | 667 | 30.1 | 16,295 | 16.8 |  |
| **Use of premenopausal progestagens** |  |  |  |  |  |
| Never | 1565 | 70.8 | 61,871 | 63.9 | <0.0001 |
| Ever | 646 | 29.2 | 34,913 | 36.1 |  |
| **Menopausal status** |  |  |  |  |  |
| Premenopausal | 1359 | 61.5 | 61,005 | 63.0 | 0.13 |
| Postmenopausal | 852 | 38.5 | 35,779 | 37.0 |  |
| **Age at menopause^c^** |  |  |  |  |  |
| <51 years | 524 | 61.5 | 21,623 | 60.4 | 0.53 |
| ≥51 years | 328 | 38.5 | 14,156 | 39.6 |  |
| **Menopausal hormone therapy use^c^** |  |  |  |  |  |
| Never | 558 | 65.5 | 19,536 | 54.6 | <0.0001 |
| Ever | 294 | 34.5 | 16,243 | 45.4 |  |
| **History of benign breast disease** |  |  |  |  |  |
| Never | 1612 | 82.9 | 70,299 | 78.0 | <0.0001 |
| Ever | 333 | 17.1 | 19,834 | 22.0 |  |
| **History of mammographic exam** |  |  |  |  |  |
| Never | 989 | 50.9 | 26,046 | 28.9 | <0.0001 |
| Ever | 956 | 49.1 | 64,087 | 71.1 |  |
| **Family history of breast cancer** |  |  |  |  |  |
| No | 2095 | 94.7 | 89,979 | 93.0 | 0.001 |
| Yes | 116 | 5.3 | 6805 | 7.0 |  |
| **UV dose in county of birth**^b^ |  |  |  |  |  |
| <1.40 | 573 | 25.9 | 26,349 | 27.2 | 0.97 |
| 1.40-1.61 | 591 | 26.7 | 27,580 | 28.5 |  |
| ≥1.61 | 618 | 28.0 | 28,727 | 29.7 |  |
| Missing | 429 | 19.4 | 14,128 | 14.6 |  |
| **UV dose in county of residence at inclusion** |  |  |  |  |  |
| <1.40 | 579 | 26.2 | 30,426 | 31.4 | 0.009 |
| 1.40-1.63 | 949 | 42.9 | 32,802 | 33.9 |  |
| ≥1.63 | 683 | 30.9 | 33,559 | 34.7 |  |

MET, Metabolic Equivalent Task; UV, Ultraviolet

^a^From chi-square tests

^b^Tests were performed excluding the unknown/missing category

^c^Among postmenopausal women
