# Supplementary material for: Association between Melanocytic Nevi and Risk of Breast Diseases: The French E3N Prospective Cohort
Source: PLoS Med. 2014 Jun 10;11(6):e1001660. doi: 10.1371/journal.pmed.1001660 (PMC4051602; doi:10.1371/journal.pmed.1001660)
Supplement: Table S3 — Hazard ratios and 95% confidence intervals for number of nevi (in four categories, considering “a few” as the reference) in relation to the risk of breast cancer, E3N cohort ( n = 89,802). (DOCX) [file pmed.1001660.s003.docx]

| **Table S3.** Hazard Ratios (HRs) and 95% Confidence Intervals (CIs) for number of nevi in relation to the risk of breast cancer, E3N cohort (n=89,802) | | | | | | | |
| --- | --- | --- | --- | --- | --- | --- | --- |
| **Number of nevi** | **n** | **Cases** | **Model 1**  **Age-adjusted HR**  **(95% CI)** | **Model 2**  **Multivariable HR^a^**  **(95% CI)** | **Model 3**  **Multivariable HR^b^**  **(95% CI)** | **Model 4**  **Multivariable HR^c^**  **(95% CI)** | **Model 5**  **Multivariable HR^d^**  **(95% CI)** |
| ***All breast cancers*** | | | | | | | |
| None | 9044 | 579 | 0.96 (0.88-1.06) | 0.98 (0.89-1.07) | 0.99 (0.90-1.08) | 0.99 (0.91-1.09) | 0.99 (0.91-1.09) |
| A few | 33,187 | 2194 | 1.00 (Reference) | 1.00 (Reference) | 1.00 (Reference) | 1.00 (Reference) | 1.00 (Reference) |
| Many | 37,911 | 2502 | 1.02 (0.97-1.08) | 1.01 (0.96-1.07) | 1.01 (0.95-1.07) | 1.01 (0.95-1.07) | 1.00 (0.95-1.06) |
| Very many | 9660 | 681 | 1.13 (1.04-1.23) | 1.11 (1.02-1.21) | 1.09 (1.00-1.19) | 1.08 (0.99-1.18) | 1.07 (0.98-1.17) |
| P_trend_ |  |  | 0.006 | 0.04 | 0.12 | 0.15 | 0.26 |
| ***In situ breast cancers*** | | | | | | | |
| None | 9044 | 61 | 0.88 (0.67-1.17) | 0.90 (0.68-1.20) | 0.91 (0.69-1.21) | 0.92 (0.69-1.22) | 0.92 (0.69-1.22) |
| A few | 33,187 | 258 | 1.00 (Reference) | 1.00 (Reference) | 1.00 (Reference) | 1.00 (Reference) | 1.00 (Reference) |
| Many | 37,911 | 297 | 1.02 (0.86-1.20) | 1.00 (0.85-1.18) | 0.99 (0.84-1.18) | 0.99 (0.84-1.18) | 0.99 (0.84-1.18) |
| Very many | 9660 | 95 | 1.30 (1.03-1.65) | 1.26 (0.99-1.60) | 1.23 (0.97-1.57) | 1.23 (0.97-1.56) | 1.22 (0.96-1.54) |
| P_trend_ |  |  | 0.03 | 0.08 | 0.12 | 0.13 | 0.15 |
| ***Invasive breast cancers*** | | | | | | | |
| None | 9044 | 518 | 0.97 (0.89-1.07) | 0.99 (0.90-1.09) | 1.00 (0.90-1.10) | 1.00 (0.91-1.11) | 1.00 (0.91-1.10) |
| A few | 33,187 | 1936 | 1.00 (Reference) | 1.00 (Reference) | 1.00 (Reference) | 1.00 (Reference) | 1.00 (Reference) |
| Many | 37,911 | 2205 | 1.02 (0.96-1.09) | 1.01 (0.96-1.08) | 1.01 (0.95-1.07) | 1.01 (0.95-1.07) | 1.00 (0.94-1.07) |
| Very many | 9660 | 586 | 1.11 (1.01-1.21) | 1.09 (0.99-1.19) | 1.06 (0.97-1.17) | 1.06 (0.97-1.17) | 1.05 (0.95-1.15) |
| P_trend_ |  |  | 0.04 | 0.11 | 0.27 | 0.33 | 0.51 |

^a^Adjusted for education, menopausal status, age at menopause (in postmenopausal women), use of menopausal hormone therapy (in postmenopausal women), use of premenopausal progestagens and stratified according to year of birth in 5-year categories

^b^Additionally adjusted for personal history of benign breast disease

^c^Additionally adjusted for family history of breast cancer

^d^Additionally adjusted for BMI, height, physical activity, age at menarche, age at first full-term pregnancy, parity, breastfeeding, use of oral contraceptives, history of mammographic exam, UV dose in county of birth and UV dose in county of residence at inclusion
