# Supplementary material for: Association between Melanocytic Nevi and Risk of Breast Diseases: The French E3N Prospective Cohort
Source: PLoS Med. 2014 Jun 10;11(6):e1001660. doi: 10.1371/journal.pmed.1001660 (PMC4051602; doi:10.1371/journal.pmed.1001660)
Supplement: Table S4 — Hazard ratios and 95% confidence intervals for number of nevi in relation to the risk of breast cancer, stratified by mean UV dose in county of birth, E3N cohort ( n = 89,802). (DOCX) [file pmed.1001660.s004.docx]

| **Table S4.** Hazard Ratios (HRs) and 95% Confidence Intervals (CIs) for number of nevi in relation to the risk of breast cancer, stratified by mean UV dose in county of birth, E3N cohort (n=89,802) | | | | | | |
| --- | --- | --- | --- | --- | --- | --- |
|  | **Mean UV dose in county of birth <1.48 kJ/m²** | | | **Mean UV dose in county of birth ≥1.48 kJ/m²** | | |
|  | ***All breast cancers*** | | | | | |
| **Number of nevi** | **n** | **Cases** | **Model 3**  **Multivariable HR^a^**  **(95% CI)** | **n** | **Cases** | **Model 3**  **Multivariable HR^a^**  **(95% CI)** |
| None | 4406 | 320 | 1.00 (Reference) | 4315 | 288 | 1.00 (Reference) |
| A few | 17,517 | 1128 | 1.02 (0.90-1.17) | 17,238 | 1183 | 1.06 (0.92-1.22) |
| Many | 14,811 | 1037 | 0.96 (0.84-1.09) | 15,710 | 1022 | 1.12 (0.98-1.29) |
| Very many | 4185 | 284 | 1.10 (0.94-1.30) | 4170 | 250 | 1.09 (0.92-1.30) |
| P_trend_ |  |  | 0.86 |  |  | 0.13 |
|  | ***In situ breast cancers*** | | | | | |
| **Number of nevi** | **n** | **Cases** | **Model 3**  **Multivariable HR^a^**  **(95% CI)** | **n** | **Cases** | **Model 3**  **Multivariable HR^a^**  **(95% CI)** |
| None | 4406 | 35 | 1.00 (Reference) | 4315 | 48 | 1.00 (Reference) |
| A few | 17,517 | 118 | 1.13 (0.75-1.72) | 17,238 | 152 | 1.05 (0.71-1.57) |
| Many | 14,811 | 117 | 0.97 (0.64-1.46) | 15,710 | 127 | 1.12 (0.75-1.66) |
| Very many | 4185 | 28 | 1.15 (0.69-1.90) | 4170 | 30 | 1.36 (0.86-2.16) |
| P_trend_ |  |  | 0.83 |  |  | 0.15 |
|  | ***Invasive breast cancers*** | | | | | |
| **Number of nevi** | **n** | **Cases** | **Model 3**  **Multivariable HR^a^**  **(95% CI)** | **n** | **Cases** | **Model 3**  **Multivariable HR^a^**  **(95% CI)** |
| None | 4406 | 285 | 1.00 (Reference) | 4315 | 240 | 1.00 (Reference) |
| A few | 17,517 | 1010 | 1.01 (0.88-1.16) | 17,238 | 1031 | 1.06 (0.92-1.23) |
| Many | 14,811 | 920 | 0.96 (0.83-1.10) | 15,710 | 895 | 1.13 (0.97-1.30) |
| Very many | 4185 | 256 | 1.10 (0.93-1.30) | 4170 | 220 | 1.05 (0.87-1.26) |
| P_trend_ |  |  | 0.79 |  |  | 0.28 |

^a^Adjusted for education, menopausal status, age at menopause (in postmenopausal women), use of menopausal hormone therapy (in postmenopausal women), use of premenopausal progestagens, personal history of benign breast disease, family history of breast cancer and and stratified according to year of birth in 5-year categories
